# Cellular versus acellular matrix devices in treatment of diabetic foot ulcers: study protocol for a comparative efficacy randomized controlled trial

**DOI:** 10.1186/1745-6215-14-8

**Published:** 2013-01-09

**Authors:** Hadar Lev-Tov, Chin-Shang Li, Sara Dahle, Roslyn Rivkah Isseroff

**Affiliations:** 1 Veterans Affairs Medical Center, Northern California Healthcare System, 10535 Hospital Way, Mather, CA, 95655, USA; 2 Department of Dermatology, University of California Davis, 3301 C Street, Sacramento, CA, 95816, USA; 3 Department of Public Health Sciences, Division of Biostatistics, University of California Davis, MS1C Room 145, Davis, CA, 95616, USA

**Keywords:** Diabetic foot ulcer, Chronic wounds, Nonhealing wounds, Oasis, Dermagraft, Wound matrix

## Abstract

**Background:**

Diabetic foot ulcers (DFUs) represent a significant source of morbidity and an enormous financial burden. Standard care for DFUs involves systemic glucose control, ensuring adequate perfusion, debridement of nonviable tissue, off-loading, control of infection, local wound care and patient education, all administered by a multidisciplinary team. Unfortunately, even with the best standard of care (SOC) available, only 24% or 30% of DFUs will heal at weeks 12 or 20, respectively.

The extracellular matrix (ECM) in DFUs is abnormal and its impairment has been proposed as a key target for new therapeutic devices. These devices intend to replace the aberrant ECM by implanting a matrix, either devoid of cells or enhanced with fibroblasts, keratinocytes or both as well as various growth factors. These new bioengineered skin substitutes are proposed to encourage angiogenesis and in-growth of new tissue, and to utilize living cells to generate cytokines needed for wound repair.

To date, the efficacy of bioengineered ECM containing live cellular elements for improving healing above that of a SOC control group has not been compared with the efficacy of an ECM devoid of cells relative to the same SOC. Our hypothesis is that there is no difference in the improved healing effected by either of these two product types relative to SOC.

**Methods/Design:**

To test this hypothesis we propose a randomized, single-blind, clinical trial with three arms: SOC, SOC plus Dermagraft® (bioengineered ECM containing living fibroblasts) and SOC plus Oasis® (ECM devoid of living cells) in patients with nonhealing DFUs. The primary outcome is the percentage of subjects that achieved complete wound closure by week 12.

**Discussion:**

If our hypothesis is correct, then immense cost savings could be realized by using the orders-of-magnitude less expensive acellular ECM device without compromising patient health outcomes. The article describes the protocol proposed to test our hypothesis.

**Trial registration:**

ClinicalTrials.gov: NCT01450943. Registered: 7 October 2011

## Background

Diabetes affects nearly one-third of the adult population in the United States [1]. For individuals with diabetes, the lifetime probability of developing a diabetic foot ulcer (DFU) is estimated at 10 to 25% [2]. DFUs represent a significant source of morbidity and an enormous financial burden [3,4]. Diabetes is the leading cause of nontraumatic lower-extremity amputations in the United States [1] and a DFU is often the initial insult leading to these amputations [5]. The pathophysiology of DFUs is thought to result from the combined comorbidities of neuropathy, vascular deficits, impaired immunity, infection and trauma, all occurring in no particular order and overlapping to produce a vicious cycle [6]. The standard of care (SOC) for DFUs involves systemic glucose control, ensuring adequate extremity perfusion, debridement of nonviable tissue, off-loading, control of infection, local wound care and patient education, all administered by a multidisciplinary team [7-10]. Unfortunately, even with the best SOC available, only 24% or 30% of DFUs will heal at weeks 12 or 20, respectively [11].

Classic wound repair has been described as a relatively linear process (albeit with significant complexity and overlap), progressing through three general phases: inflammatory, including platelet aggregation, release of proinflammatory cytokines and recruitment of neutrophils and macrophages; proliferative, including fibroblast proliferation, angiogenesis and formation of granulation tissue and keratinocyte proliferation and re-epithelialization; and remodeling, the longest phase, including myofibroblast transformation and slow restructuring of the healed wound to increase its strength [6,12].

In nonhealing DFUs the normal healing process is stalled. The exact mechanisms involved in this impairment are not fully understood but an increasing number of pathways are suggested with varying levels of evidence and are summarized herein as reviewed in a number of recent excellent articles [13-18]. Hyperglycemia is thought to adversely affect healing by increased levels of advanced glycation end products that inhibit normal extracellular matrix (ECM) deposition and upregulate activity of matrix metalloproteinases (MMPs). Prolonged hyperglycemia may also offset the delicate balance between reactive oxygen species and various antioxidants by depletion of nicotinamide adenine dinucleotide phosphate on one hand and increasing reactive oxygen species production on the other. DFUs have increased activity of MMPs coupled with decreased activity of MMP inhibitors (tissue inhibitors of metalloproteinases). Fibroblasts in DFUs are often senescent, exhibit decreased proliferation and are less responsive to growth factors when compared with fibroblasts from age-matched diabetic controls without ulceration. Keratinocytes in diabetic wounds exhibit impaired migration, which may be mediated by c-Myc and β-catenin. A drastic decrease in available inorganic phosphate leads to decreased levels of ATP in DFUs – a devastating blow to all healing-related processes. Immune response is impaired, with reduced inflammatory cell recruitment at the initial phase and later a skewed immune response towards macrophage and B-cell infiltrates. That phase is also characterized by increased TNFα and IL-1β expression, which are known MMP stimulators. Leukocyte function and intracellular killing are impaired in DFUs, rendering these ulcers exceptionally susceptible to superficial infections and resistant biofilms. Neuropathy leads to decreased levels of neuropeptides that normally contribute to healing. In addition, neuropathy reduces capillary blood flow. Impaired gap junction function has recently immerged as an additional pathological mechanism leading to impaired wound healing in DFUs.

As described above, the ECM is abnormal in DFUs and its impairment has been proposed as a key target for new therapeutic devices [19]. These devices intend to replace the aberrant ECM by implanting a matrix, either devoid of cells or enhanced with fibroblasts, keratinocytes or both as well as various growth factors. These new bioengineered skin substitutes are proposed to encourage angiogenesis and in-growth of new tissue, and to utilize living cells to generate cytokines needed for wound repair. Dermagraft® (Shire Regenerative Medicine, Inc. La Jolla, California, United States ) is one example of bioengineered matrix supplemented with fibroblasts (cellular matrix (CM)). Other “new-generation” ECM replacement tissues, devoid of cellular components, have been created for wound repair, and the Oasis® (Healthpoint, Ltd Fort Worth, Texas, United States ) acellular matrix (ACM) is an example of this category. Interestingly, in industry-supported randomized controlled trials, the reported rates of wound closure at week 12 are approximately 50% for both devices [20,21]. A success rate of 50% represents about 20% added benefit to the use of such devices over the SOC. This minimal benefit may be disproportionate to the expenditure these devices accumulate (for example, up to $1,800 per application, and up to eight applications as the recommended regimen).

The efficacy of bioengineered ECM containing live cellular elements (CM) for improving healing above that of a SOC control group has not to date been compared with the efficacy of an ECM devoid of cells (ACM) relative to the same SOC. Our hypothesis is that there is no difference in the improved healing effected by either of these two product types relative to the SOC. If this hypothesis is correct, then immense cost savings could be realized by using the orders-of-magnitude less expensive acellular ECM device without compromising patient health outcomes. To test this hypothesis we proposed a randomized, single-blind, clinical trial with three arms: SOC, SOC plus Dermagraft® (bioengineered ECM containing living fibroblasts) and SOC plus Oasis® (ECM devoid of living cells) in patients with nonhealing DFUs.

## Methods/Design

### Participants

The study protocol is approved by both the Veterans Affairs’ Institutional Research and Development Committee and their Institutional Review Board (IRB). Study participants are veterans eligible for Veterans Affairs’ medical benefits. Patients are recruited from all clinics at the Veterans Affairs’ Northern California Healthcare System via provider referral and IRB-approved flyers. The study is supported by a Veterans Affairs’ MERIT award (project ID SURG-369-10S). During the “run-in” phase (see Table 1) all participants are screened for eligibility based on the inclusion and exclusion criteria. All study-related procedures (except diagnostic tests such as radiography or blood testing) are performed by study staff at participating Veterans Affairs Northern California Healthcare System wound centers.

**Table 1:** Study procedure schedule

**Procedure**	**Visit 1 (week −2)**	**Visit 2 (week −1)**	**Visit 3 (week 0), randomization**	**Visits 4 to 10 (weeks 1 to 7), treatment (variable)**	**Visits 11 to 14 (weeks 8 to 11), treatment**	**Visit 15 (week 12), study endpoint**	**Visits 16 to 19 (weeks 13 to 28), follow-up**
Informed consent	X						
Inclusion/exclusion criteria	X	X	X				
Physical examination	X					X	
Vital signs	X	X	X	X	X	X	X
Ankle-brachial index	X						
Fungal infection culture and malignancy evaluation	X						
Blood sample drawn for laboratories	X					X	
SF-36v2™ questionnaire	X					X	
Ulcer assessment	X	X	X	X	X	X	X
Debridement	X	X	X	X	X	X	X
Ulcer photography and area measurement	X	X	X	X	X	X	X
Randomization			X				
Dermagraft® Tx + SOC			X	X			
Oasis® Tx + SOC			X	X			
SOC and off-loading	X	X	X	X	X	X	X
Concomitant medications and adverse events	X	X	X	X	X	X	X

Dermagraft® (Advanced BioHealing, Inc. La Jolla, California, United States), Oasis® (Healthpoint, Ltd, Fort Worth, Texas, United States). SF-36v2™, Short Form-36, version 2; SOC, standard of care; Tx, treatment.

### Inclusion criteria

• Institutional Review Board (IRB) Informed Consent Form is signed and dated prior to any study-related activities.

• Area of the study ulcer after debridement is between 1 and 25 cm^2^ at week 0/visit 3.

• Subject is between 18 and 85 years of age.

• Subject's highest Ankle Brachial Pressure Index (ABPI)/Ankle Arm Index (AAI) ≥ 0.80 and < 1.4 (Highest ABPI/AAI value from three measurements within last 6 months shall apply) or toe–arm index ≥ 0.6.

• Patient has one or more diabetic ulcers on the target foot with only one ulcer selected as the study (target) ulcer. The target ulcer must be at least 4 cm from a nontarget ulcer and, in the investigator’s opinion, be unlikely to coalesce with another ulcer within 12 weeks of randomization.

• Subject's study ulcer is full thickness and does not extend to bone, muscle, or tendon.

• Subject's study ulcer has been present at least 4 weeks prior to the initial screening (visit 1) or 6 weeks at randomization (visit 3).

• Subject has been diagnosed with type 1 or type 2 diabetes and hemoglobin A1c < 10%.

• Study ulcer has no clinical feature of infection (two signs of inflammation and elevated bacterial load of the wound).

• Female subjects of childbearing age potential have a negative pregnancy test and are lactating for the duration of the study.

• Subject understands the requirements of this study and is willing to comply with all the study requirements.

### Exclusion criteria

• Subject is diagnosed with cancer and is undergoing treatment with immunosuppressive or chemotherapeutic agents, radiotherapy or systemic corticosteroids < 30 days before enrollment.

• Subject is diagnosed with HIV/AIDS.

• Subject is diagnosed with any bleeding disorders.

• Subject is diagnosed with any connective tissue diseases.

• For female subjects, the subject is pregnant or lactating.

• Subject has a history of illicit drug use within 1 year of enrollment.

• In the past year, the subject experienced episodes of drinking more than five alcoholic beverages in <2 hours and/or drinking alcohol became a problem in their interpersonal relationships, work, driving and/or their behavior in general.

• Subject has any active infected wounds or osteomyelitis (confirmed by bone biopsy, magnetic resonance imaging or bone scan).

• Subject had Doppler examination within the last 365 days, and it demonstrated reflux > 0.5 seconds.

• Subject is diagnosed with active Charcot as described by Saunder’s classification system.

• Subject manifests signs of poor nutritional status and/or albumin level < 2.9 g/dl.

• Subject received Dermagraft® and/or Oasis® in the last 60 days.

• Study ulcer size < 1.0 cm^2^ or >25 cm^2^.

• Subject has any porcine allergy or cow product allergy.

• Subject’s recent (last 30 days) chemistry test’s serum creatinine is two times above the upper limit of normal and/or liver enzymes (AST, ALT) are three times above the upper limit of normal.

• Between week −2/visit 1 and week 0/visit 3 (randomization) the study ulcer decreased in size by > 40%, or increased in size by > 50%.

Dermagraft® (Advanced BioHealing, Inc. La Jolla, California, United States), Oasis® (Healthpoint, Ltd Fort Worth, Texas, United States).

### Participant consent

Detailed informed consent is obtained from all participants and a HIPAA Health Insurance Portability and Accountability Act release form is signed. Prior to any study-related intervention, study staff provide each prospective subject with a detailed explanation of the nature and purpose of the trial. Once study staff are assured that the prospective subject understands all study procedures and potential implications, an IRB-approved informed consent form is signed. The signed copy becomes part of the subject’s record. An additional copy of the informed consent is provided to the subject for future reference.

### Criteria for withdrawal

Subjects are informed that they may withdraw from the trial for any reason at any time and that the investigator may decide to withdraw them from the trial for safety-related issues. Study staff explain that failure to comply with the study’s procedure may result in withdrawal from the trial. In the case of such an event, the reason for withdrawal is documented in the subject’s file and the subject is referred to the wound clinic for further follow-up.

### Participant compliance

Overall participant compliance with study-related procedures is assessed at each study visit by the staff. Specifically, detailed questioning regarding compliance with off-loading and any tampering with study-related dressings is performed at each study visit and any deviations are noted in the subject’s file for later analysis.

### Summarized details of products compared (interventions)

The CM of this study, Dermagraft®, is a cryopreserved human fibroblast-derived dermal substitute composed of viable newborn foreskin fibroblasts, seeded onto a bioabsorbable polyglactin mesh. The product is supplied frozen (−75°C) in a clear bag containing a 2 inch×3 inch matrix [22]. The product is stored according to the manufacturer’s instructions and records of quality control, including constant temperature monitoring, are kept by study staff. Dermagraft® is applied according to the manufacturer’s instructions [22].

The ACM of this study, OASIS®, is a bioabsorbable ECM derived from porcine small intestinal submucosa. The product is processed using proprietary methods to retain its original structure while stored at room temperature [23]. The product is stored and applied according to the manufacturer’s instructions [23].

The SOC dressing is comprised of Iodosorb® gel (Smith & Nephew Largo, Florida, United States), Adaptic® (Johnson & Johnson Gargrave, Skipton, United Kingdom ) and gauze. This dressing also serves as the secondary dressing for the two intervention arms. If a subject has an allergy to iodine products, then Bacitracin antibiotic ointment is used as a replacement for Iodosorb.

We will provide all subjects with a removable walking boot, such as a camwalker or diabetic conformer Bledsoe boot. The clinician will make further accommodation/adjustment to offload plantar ulcers with excavation or aperature using plastazote, foam or felt padding. Those patients that use exclusively manual or motorized wheelchairs will be identified to differentiate patients that are fully ambulatory from those with limited ambulatory capacity. While total contact casting is considered the gold standard for offloading plantar ulcers by many academicians, in our experience total contact casting is not in standard use owing to time constraints with cast application and accessibility to materials. Indeed, a recent study found that fewer than 2% of diabetic foot specialists utilize this form of offloading [24]. Our study therefore models the standard care for all arms after realistic clinical practice.

All products used in this trial are approved by the Food and Drug Administration for use in treatment of DFUs.

### Hypothesis (objectives)

There is no significant difference in healing at weeks 12 and week 20 or in the rate of healing when treating DFUs with SOC as compared with CM or ACM.

### Alternative hypothesis

There is a significant difference in healing at weeks 12 and week 20 or in the rate of healing when treating DFUs with SOC as compared with CM or ACM.

### Primary outcome

The primary outcome is the percentage of subjects that achieved complete wound closure by week 12. Complete healing is defined as full re-epithelialization of the ulcer with no drainage, or callus formation with underlying ulcer on two consecutive visits 1 week apart. Callus (hyperkeratosis) formation will be debrided at the wound site to determine whether complete closure has been obtained. The wound is deemed healed if there is no underlying open lesion after debridement (complete epithelialization).

### Secondary outcomes

The first secondary outcome is the percentage of subjects that achieved complete wound closure by week 20. Complete healing will be defined as full re-epithelialization with no drainage, or callus formation with underlying ulcer on two consecutive visits 1 week apart.

Another secondary outcome is the rate of wound healing to achieve complete closure based on weekly wound area measurements.

Third is the incidence of ulcer recurrence at week 20. Recurrence will be defined as an ulcer occurring at the same location as the healed study ulcer.

Fourth is the association of wound healing (overall and given a particular treatment) with the following wound characteristics: peri-ulcer erythema, induration, tenderness, pain, local warmth, size, depth, undermining, ulcer location, ulcer age at presentation, ulcer precipitating event and total wound assessment score at presentation.

Another outcome is the association of wound healing (overall and given a particular treatment) with the following patient characteristics on presentation: age, sex, body mass index, smoking history, family history of diabetes, complete blood count results, chemistry panel results, erythrocyte sedimentation rate and C-reactive protein serum concentration and hemoglobin A1C serum concentration.

Sixth is the association of a particular treatment with the incidence of the following during the study period: cellulitis, osteomyelitis, acute Charcot disease and overall adverse events rate.

Another outcome is the association of a particular treatment with a change in quality of life assessments (quality-adjusted life-years).

The final secondary outcome is cost-effectiveness of a particular treatment compared with SOC.

### Sample size

Based on previous trials [20,21] it is estimated that approximately 50% of the subjects in the intervention arms will achieve complete wound closure at week 12 (primary outcome) and approximately 25% of the SOC arm will achieve complete wound closure by week 12. The sample size was estimated based on 80% power and a 0.05 significance level to detect a 25% difference between the intervention and control arms (Stplan 4.5 statistical software, Department of Biomathematics, University of Texas MD Anderson Cancer Center, Houston, TX, USA, 2010). Based on the method of normal approximation to the arcsin transformation of the binomial distribution and, to be conservative, a two-sided test, enrollment of 57 subjects in each arm is required.

### Randomization

#### Sequence generation

Since healing has been shown to depend on age and race [25,26], randomization was done in small blocks (six subjects per block) in order to match subjects according to age groups (< 50 years old, 50 through 59 years old, 60 through 69 years old and 70 through 85 years old) and race (black and nonblack). We used a computer-generated algorithm to randomly assign treatments to subjects within a block and to randomly determine the block sequence. Subjects presenting to the study will be entered into their appropriate block in a strict chronological order.

#### Allocation concealment and implementation

The randomization table was generated prior to study initiation by the study’s biostatistician. Allocation is concealed by the Northern California Veterans Affairs Investigational Drug Service, an independent third party, until the moment of randomization.

### Blinding

Owing to the nature of the interventions studied, complete blinding is not realistic (for example, Dermagraft® requires storage at −75 ± 10°C and a specific manipulation immediately prior to application). An independent observer was therefore assigned to assess the primary and secondary outcomes. Clinical images will be captured at each visit before and after debridement and will be de-identified and uploaded to an external server (Silhouette Central® Aranz Medical, Christchurch, New Zealand). The observer will log on to the server independently and determine whether the wound has closed or not. In addition, the observer will determine whether the margins used for area measurement are accurate.

### Statistical methods

All data will be analyzed based on intention to treat. The primary outcome, wound closure by week 12, will be analyzed using the chi-square test or Fisher’s exact test when appropriate to compare the percentages of subjects with complete closure in each group. We will also perform exploratory analysis with logistic regression, using complete wound closure at 12 weeks as the dependent variable and the independent variables would include the characteristics described as the fourth to sixth items in Secondary outcomes above. We will run preliminary analysis on selected covariates to determine whether there are significant differences.

Cost-effectiveness analysis will be performed to compare the treatment groups Dermagraft® and Oasis® and the standard care group using the analysis of variance *F* test or Kruskal–Wallis test according to the data distribution pattern. If significant, we will further analyze with pair-wise comparisons using Tukey’s test or Dunn’s test. A multiple-comparison *post-hoc* test for Kruskal–Wallis analysis will be performed with the Statistical Analysis System (Cary, NC, USA) macro developed by Elliott and Hynan [27].

We will evaluate the cost-effectiveness of Oasis® compared with Dermagraft® and the SOC by measuring quality-adjusted life-years as the measure of effectiveness, based on results obtained from Short Form-36 (SF-36v2™) questionnaires. Multiple regression will be used to evaluate continuous dependent variable outcomes, including change in SF-36v2™ physical and mental component summaries scores between baseline and the end of the study.

Rates of healing among the groups will be analyzed using a log-rank test to compare the time of healing within 20 weeks.

Secondary outcomes such as complete healing at 20 weeks and rate of ulcer recurrence at 20 weeks will be analyzed using chi-square tests or Fisher’s exact tests when appropriate. In addition, demographics, smoking history, and other characteristics mentioned in Secondary outcomes will be summarized in a table. For comparison of nominal categorical secondary outcome variables, we will use the chi-square test or Fisher’s exact test when appropriate. For comparison of ordinal categorical variables, we will use the Wilcoxon rank-sum test (for two independent group comparisons) and the Kruskal–Wallis test (for three independent group comparisons).

### Data management

Data will be managed by the study’s biostatistician (C-SL) at the University of California Davis Clinical and Translational Science Center. Data will be automatically imported from digital subject records into pre-designed spread sheets using Excel® and analyzed using Statistical Analysis System, version 9.3 [28].

### Plan and trial design

This is a randomized, controlled, single-blind, trial comparing the efficacy of ACM with that of CM in treatment of DFUs. A total of 171 subjects will be enrolled and randomly assigned to one of three treatment groups: ACM, CM and SOC. Subjects are followed for a total of 30 weeks in three major phases: run-in phase, treatment phase and follow-up phase. The study’s procedures are detailed below.

#### Run-in phase

During this 2-week period (weeks −2 through 0) prospective subjects are seen on a weekly basis and rigorously evaluated for eligibility. Once informed consent is secured, detailed health-related history is solicited and a thorough physical examination is performed. Comprehensive lower-extremity ulceration evaluation is done (including imaging and area measurements) and compliance with an off-loading device is assessed. The following laboratory studies are ordered to exclude comorbidities and establish a baseline: vital signs, body mass index, pregnancy test, Ankle-Brachial Index, quantitative bacterial cultures, fungal cultures, tissue pathology, complete blood count, comprehensive metabolic panels (including liver enzymes and albumin levels), erythrocyte sedimentation rate, C-reactive protein level, hemoglobin A1C level and lower-extremity X-rays. Once eligibility is established, subjects are randomized (as described above) to one of three treatment arms. SF-36 questionnaires are filled out by the subjects.

#### Treatment phase

This phase is divided into a variable treatment period (weeks 0 through 7) and a SOC treatment period (weeks 8 through 11). Subjects will be evaluated on a weekly basis.

During the variable treatment period, subjects in each arm will receive its corresponding primary dressing treatment (that is, ACM, CM or SOC). During each visit, vital signs will be measured, a comprehensive lower-extremity assessment will be performed (including photography and ulcer area measurement), health status and medication changes will be recorded and adverse events will be assessed. Compliance with the off-loading device will also be assessed.

During the SOC treatment phase the primary dressing applied to all subjects will be identical and will comprise the SOC dressing. The rest of the visit will be identical to the variable treatment period as described above.

On week 12, a study endpoint visit will be conducted. During this visit, vital signs will be measured, a comprehensive lower-extremity assessment will be performed (including photography and ulcer area measurement), health status and medication changes will be recorded and adverse events will be assessed. Compliance with the off-loading device will also be assessed. In addition, the SF-36 questionnaire is filled out for the second time by the subjects and repeat complete blood count and comprehensive metabolic panels are ordered. SOC treatment will be provided to all subjects.

#### Follow-up phase

This phase is comprised of four monthly visits (weeks 13 through 28). During each visit, vital signs are measured, a comprehensive lower-extremity assessment is performed (including photography and ulcer area measurement), health status and medication changes are recorded and adverse events are assessed. Compliance with the off-loading device is also assessed. During the follow-up phase the primary dressing applied to all subjects is identical and will comprise the SOC dressing. SOC treatment is provided to all subjects.

In the event of early wound closure (before week 12), the subject will return for follow-up visits every 4 weeks from the confirmatory visit and in week 12 or in week 12 only, whichever comes first, and will return to the schedule above thereafter. In the event of failure to heal after week 12, subjects will be followed at the wound clinic based on clinical need but will return for all follow-up phase mandated visits.

### Ethical considerations

The DOLCE trial is approved by the Veterans Affairs Northern California Healthcare System IRB and the Research and Development Committee. The primary investigators will ensure that the study is conducted in full compliance with the protocol and IRB regulations as well as international standards on human subjects’ research. The primary investigators will ensure compliance with institutional regulations as well as local and national law. Compliance will be administered by a dedicated, certified Clinical Research Coordinator. A data monitoring committee has been set up to review safety data. All adverse events will be reported to the IRB as stipulated by the IRB in its protocol-approval letter.

## Discussion

The DOLCE trial is a randomized, single-blind, comparative efficacy trial to assess the difference between ACM, CM and SOC in the treatment of DFUs. As the healthcare-related industry and science are marching towards development of increasingly complex and expensive devices, it is crucial to regularly and rigorously reassess the efficacy of various available interventions. Healthcare providers are constantly presented with new devices and need solid evidence to support clinical decision-making. However, many studies designed to provide such evidence are industry supported, and thus subject to concerns regarding inherent bias – if not with the design and execution of the trial, then certainly with the dissemination of the results, should those results not favor the product whose manufacturer sponsored the trial ([29]). In addition, in this study, subanalysis may allow for a better understanding of the indications for preferring one device over the other. Practicing within financial constraints is especially important when treating a prevalent condition such as DFUs. Providing the wound care specialist with the data to support the use of less expensive treatments without compromising patient outcomes could potentially save the healthcare system significant resources. The DOLCE trial is an attempt to provide wound care specialists with relevant evidence to an overwhelming clinical problem in order to support meaningful clinical decision-making. In addition, we hope to set an example for the importance of independent, investigator-initiated comparative efficacy research.

### Trial status

Actively recruiting.

## Abbreviations

ACM: acellular matrix; CM: cellular matrix; DFU: diabetic foot ulcer; ECM: extracellular matrix; IL: interleukin; IRB: Institutional Review Board; MMP: matrix metalloproteinase; SF-36: Short Form-36; SOC: standard of care; TNF: tumor necrosis factor.

## Competing interests

The authors declare that they have no competing interests.

## Authors’ contributions

HL-T contributed to the protocol design, wrote IRB amendments, and wrote the first draft of the manuscript. C-SL contributed to the statistical design and edited the manuscript. SD contributed to the overall study design, wrote the first draft of the protocol, edited the manuscript, and wrote the MERIT grant. RI conceived the study idea, supervised the study design and protocol development, edited the manuscript, edited IRB documents, and wrote the MERIT grant. All authors read and approved the final manuscript.
